# Supplementary material for: Glycaemia Fluctuations Improvement in Old-Age Prediabetic Subjects Consuming a Quinoa-Based Diet: A Pilot Study
Source: Nutrients. 2022 Jun 1;14(11):2331. doi: 10.3390/nu14112331 (PMC9183167; doi:10.3390/nu14112331)

Supplementary

Table S1. Comparison of nutritional values of products marketed in a regular diet with similar ingredients than products based on quinoa for the study.

	Energy		Proteins		Fats		Carbohydrates	
	RD	QD	RD	QD	RD	QD	RD	QD
Cookie	456	366,43	6,14	9,11	21,05	16,15	63,1	49,5
Cracker	400	352,83	10	9,75	10	16,9	65	35,54
Brioche	205	278,69	7,69	9,72	2,56	7,87	38,46	39,23
Sponge cake	388	300,14	6,25	11,49	16,25	18,18	53,75	24,55
Sliced bread	344	202,87	6,25	6,86	12,5	7,66	50	24,94
Baguette bread	263	210,35	7,02	6,95	1,75	4,96	50,88	29,63
Pasta	357,14	323,45	12,5	12,12	1,79	12,3	75	35,68

gr/100gr of product

The nutritional information of the products in RD was made choosing equivalent marketed products in ingredients used with the difference of the origin of the flour

Table S2. Baseline characteristics of 9 patients participating in the study.

Patient	Sex	Age	IFG	Weight	BMI	HbA1c	SBP	DBP	Waist /hip	HR	Fam. hist. DM	CV	HLD	HTA	Smoker	Alcohol
num	M / F	Years	Years	Kg	Kg / m2	%	mmHg	mmHg	Ratio	bpm	Y / N	Y / N	Y / N	Y / N	Y / N / E	M / D / N
1	M	69	2	77	26	5.8	144	90	0.92	63	N	N	Y	Y	Y	M
2	F	73	3	72	34	5.9	159	85	0.82	70	Y	N	N	N	N	M
3	F	67	Na	75	31	5.9	126	85	0.95	83	N	Y	Y	Y	Y	M
4	F	66	2	64	25	6.1	135	95	0.79	66	Y	N	Y	Y	N	D
5	M	69	3	90	31	6.7	125	75	0.93	48	N	N	N	N	N	M
6	F	74	3	69	27	6.2	107	65	0.87	67	N	N	N	Y	N	M
7	F	67	3	82	30	6.5	130	85	0.83	67	N	N	N	Y	N	N
8	M	72	0	76	27	5.7	129	58	0.92	59	N	N	N	N	Y	M
9	F	69	2	61	25	6.3	120	80	0.89	66	Y	N	Y	Y	N	D
n = 9	3M / 6F	69.6 (2.8)	2.3 (1.0)	74.0 (8.7)	28.4 (3.2)	6.1 (0.3)	130.4 (14.6)	79.6 (11.9)	0.88 (0.06)	65.3 (9.3)	3Y / 6N	1Y / 8N	4Y / 5N	6Y / 3N	3Y / 6N	6M / 2D / 1N

Num, number; M / F, male / female; bpm, beats per minute; Y / N, yes / no; Y / N / E, yes / no / ex-smoker; M / D / N, monthly / daily / no; IFG, time on impaired fasting glucose; BMI, body mass index; SBP, systolic blood pressure; DBP, diastolic blood pressure; HR, heart rate; fam, family; hist, history; DM; diabetes mellitus; CV, cardiovascular disease; HLD, hyperlipidemia; HTA, hypertension; Alcohol, alcohol consumption; na, not available.

Age, IFG, weight, BMI, HbA1c, SBP, DBP, waist/hip and HR are expressed as mean (standard deviation).

Table S3. Dietary intake differences between regular and quinoa diet.

Variables	Regular diet	Quinoa diet	*Pvalue*
	Median (Q1, Q3)	Median (Q1, Q3)	
Carbohidrats, g	32.1 (29.6, 37.8)	28.3 (22.8, 29.4)	0.004
Vegetable fiber, g	5.3 (4.9, 6.1)	4.5 (3.8, 4.8)	0.039
Lipids, g	15.3 (14.4, 16.1)	19.9 (18.8, 20.8)	0.004
SFAs, g	4 (3.9, 5.3)	5.1 (4.4, 5.8)	0.012
MUFAs, g	6.8 (6.3, 7.5)	9.4 (8.7, 9.6)	0.027
Sodium, mg	386.3 (352.9, 554.6)	335.1 (299.1, 389.8)	0.020
Vitamin B2, µg	0.32 (0.31, 0.35)	0.4 (0.39, 0.41)	0.008
Starch, g	18.6 (15.4, 20.5)	6.3 (5.6, 8.9)	0.004
Myristic acid (C14:0), g	0.34 (0.25, 0.41)	0.44 (0.33, 0.52)	0.012
Palmitoleic (C16:1), g	0.23 (0.22, 0.26)	0.28 (0.27, 0.3)	0.008
Oleic (C18:1), g	6.5 (5.9, 7)	8.8 (8.2, 8.9)	0.020
Folic acid, µg	0.22 (0, 0.43)	0 (0, 0)	0.043
Vitamin E, mg	1.8 (1.5, 2)	2.2 (2, 2.3)	0.004
Tocopherol total, mg	1.3 (1.2, 1.3)	1.7 (1.6, 1.8)	0.004
α - Tocopherol, mg	0.98 (0.88, 1.05)	1.27 (1.24, 1.43)	0.004
β - Tocopherol, mg	0.01 (0.01, 0.01)	0.02 (0.01, 0.02)	0.004
γ - Tocopherol, mg	0.3 (0.2, 0.41)	0.55 (0.5, 0.69)	0.004
δ - Tocopherol, mg	0.03 (0.02, 0.04)	0.06 (0.05, 0.07)	0.008
Zinc, mg	1.9 (1.7, 2.1)	2.2 (2, 2.3)	0.039
Magnessium, mg	0.62 (0.61, 0.68)	0.47 (0.38, 0.49)	0.012
Fluoride, µg	57.4 (52.4, 65.1)	46.3 (43.9, 59.6)	0.027
Selenium, µg	19.6 (18.7, 21.7)	14.1 (12.1, 19.5)	0.039
Cystine, mg	170.6 (164.2, 179.6)	146.3 (142.5, 167.5)	0.008
Arginine, mg	699.7 (688.3, 721.3)	591.7 (571.9, 666.4)	0.008
Glutamic acid, mg	2339 (2177.6, 2540)	1981.4 (1847.3, 2228.8)	0.008

cis-MUFAs, g	5.1 (4.6, 5.7)	6.6 (4.8, 7.8)	0.027
trans-PUFAs, g	0.01 (0.01, 0.03)	0.02 (0.02, 0.04)	0.039
Cellulose, g	0.73 (0.68, 0.87)	0.63 (0.56, 0.64)	0.039
Polysaccharides, g	0.65 (0.56, 0.88)	0.47 (0.39, 0.51)	0.020
Phytic acid, g	0.02 (0.01, 0.04)	0 (0, 0.01)	0.027
Proline, mg	755.7 (715, 893.8)	667.7 (637.8, 734.6)	0.039
Glycemic index	47 (44, 48.9)	37.3 (35.3, 40.8)	0.004
Glycaemic load	15.9 (13.3, 16.8)	6.9 (6.2, 9.8)	0.004
ORAC	2365.2 (2296.4, 2584.1)	1881.4 (1582.3, 2513.1)	0.025
Nitrogen	1.3 (1.1, 13.5)	61.8 (52, 97.6)	0.004
% of energy from lipids	32.1 (30.9, 34)	41.3 (36.7, 43.4)	0.004
% of energy from carbohidrats	47.2 (41.9, 49)	36.5 (35.4, 40.2)	0.004

SFAs, saturated fatty acids; MUFAs, Monounsaturated fatty acids; cis-MUFAs, cis-monounsaturated fatty acids; trans-PUFAs, trans-polyunsaturated fatty acids; ORAC, oxygen radical absorbance capacity.

Values are presented in median and quartiles. For each participant, mean value for dietary intake, including all meals, was considered.

Variables correspond to the nutrients with statistically significant differences ($p \leq 0.05$ using the Wilcoxon signed rank test) between two groups.

Figure S1. Diagram participant flow.

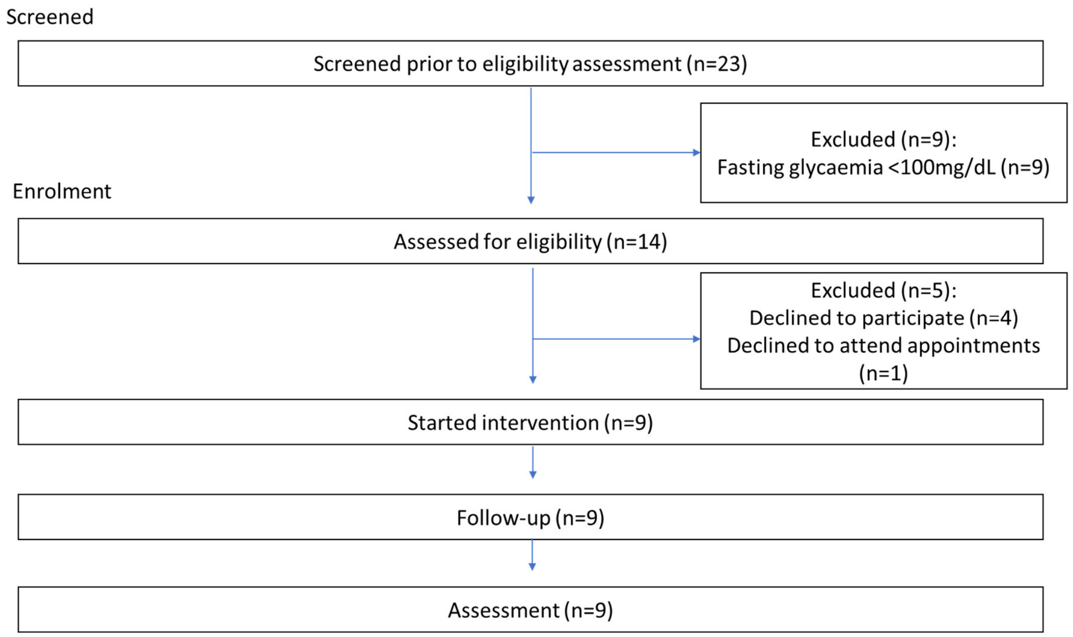

According to Eldridge et al. CONSORT 2010 statement: extension to randomised pilot and feasibility trials. Pilot and Feasibility Studies (2016) 2:64

Figure S2. Glucose concentrations of patients (a) at discrete time points (b) at equal time points with interpolation (c) as aligned curv

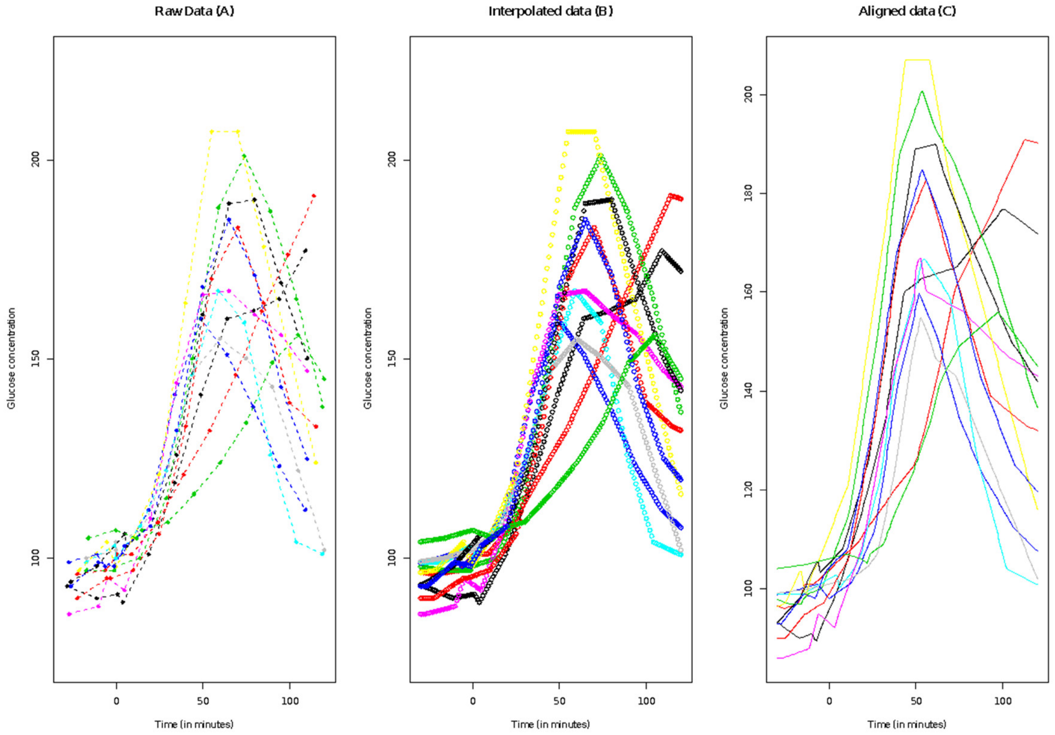

Supplement: Supplementary file 1 [file nutrients-14-02331-s001.zip › nutrients-1665221-supplementary.pdf]
